# Advancements and future prospects of adeno-associated virus-mediated gene therapy for sensorineural hearing loss

**DOI:** 10.3389/fnins.2024.1272786

**Published:** 2024-01-24

**Authors:** Linke Li, Tian Shen, Shixi Liu, Jieyu Qi, Yu Zhao

**Affiliations:** ^1^Department of Otorhinolaryngology Head and Neck Surgery, West China Hospital, Sichuan University, Chengdu, China; ^2^State Key Laboratory of Bioelectronics, Department of Otolaryngology Head and Neck Surgery, Zhongda Hospital, School of Life Sciences and Technology, Advanced Institute for Life and Health, Jiangsu Province High-Tech Key Laboratory for Bio-Medical Research, Southeast University, Nanjing, China

**Keywords:** sensorineural hearing loss, adeno-associated virus, gene therapy, delivery route, dual-AAV

## Abstract

Sensorineural hearing loss (SNHL), a highly prevalent sensory impairment, results from a multifaceted interaction of genetic and environmental factors. As we continually gain insights into the molecular basis of auditory development and the growing compendium of deafness genes identified, research on gene therapy for SNHL has significantly deepened. Adeno-associated virus (AAV), considered a relatively secure vector for gene therapy in clinical trials, can deliver various transgenes based on gene therapy strategies such as gene replacement, gene silencing, gene editing, or gene addition to alleviate diverse types of SNHL. This review delved into the preclinical advances in AAV-based gene therapy for SNHL, spanning hereditary and acquired types. Particular focus is placed on the dual-AAV construction method and its application, the vector delivery route of mouse inner ear models (local, systemic, fetal, and cerebrospinal fluid administration), and the significant considerations in transforming from AAV-based animal model inner ear gene therapy to clinical implementation.

## Introduction

Hearing loss (HL) is a common health condition affecting over 5% of the global population, approximately 430 million. Projections estimate that by 2050, more than 700 million people, or one in every 10, will experience disabling HL (World Health Organization, 2023). HL is typically categorized into two principal categories: conductive HL, which stems from problems with the ear canal, eardrum, or middle ear and its tiny bones, and sensorineural HL (SNHL), which arises from damage to either the cells in the cochlea or the auditory nerve itself. SNHL can be congenital and present from birth due to genetic factors or infections during pregnancy, such as rubella. Alternatively, it can be acquired after birth and can develop at any stage in life due to various factors such as noise-induced injury, ototoxic drugs, aging, infections like meningitis or mumps, and conditions like Meniere’s disease. Currently, the primary management strategies for SNHL involve using hearing aids or cochlear implants. These interventions benefit many but do not cure the underlying condition causing the HL. Adeno-associated virus (AAV)-mediated gene therapy represents a novel frontier in the management of SNHL. This method diverges from the traditional use of hearing aids and cochlear implants, which amplify or directly stimulate the auditory nerve to enhance auditory perception. Instead, AAV-mediated gene therapy employs AAV vectors to introduce therapeutic agents into the inner ear, thereby addressing the genetic underpinnings of HL. At the preclinical trial stage, this innovative approach has demonstrated promising potential in treating both congenital and acquired forms of SNHL.

## Anatomy and function of the inner ear

**Figure 1:**
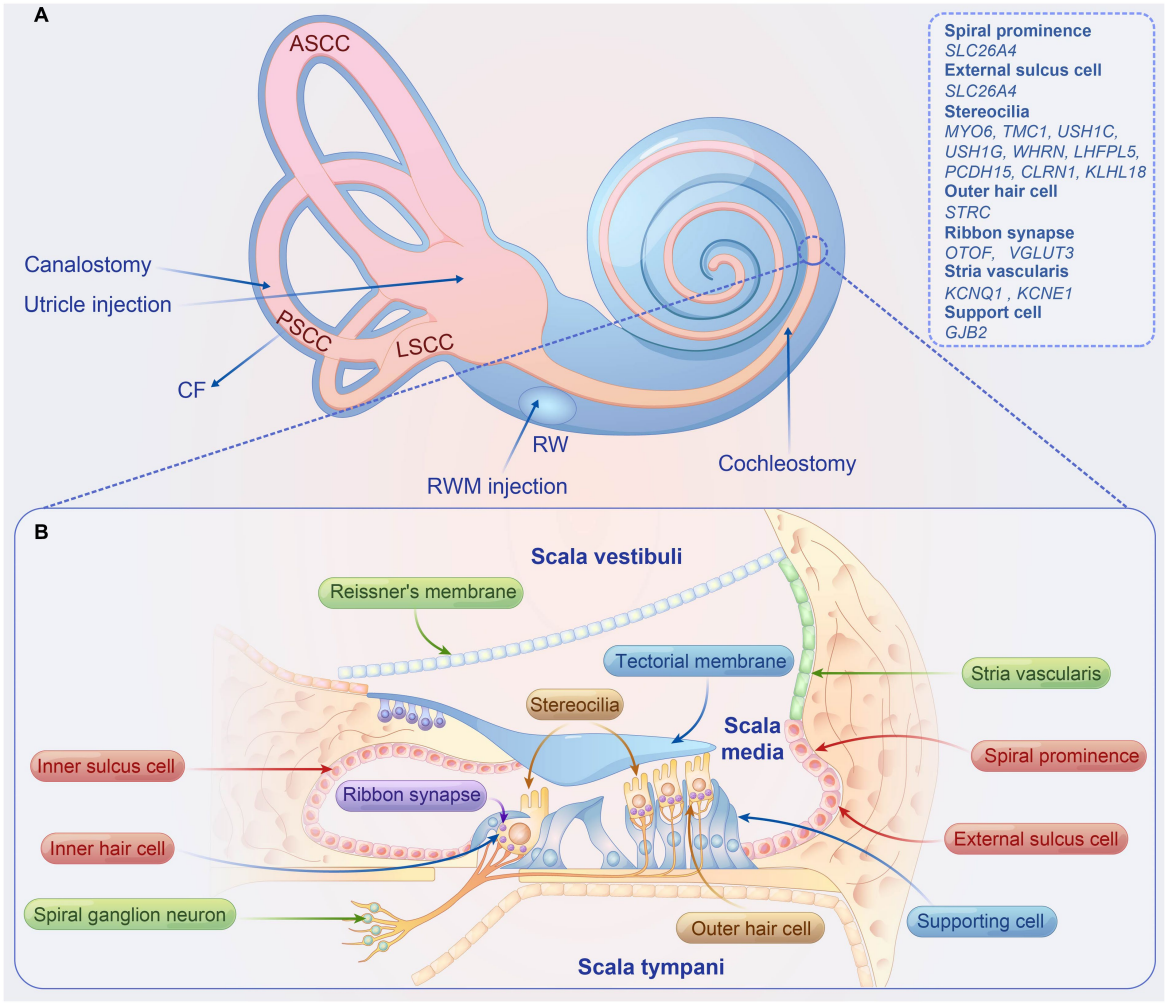
Overview of inner ear delivery routes, structure of the organ of corti, and causal genes associated with hearing loss in preclinical studies. **(A)** The four local delivery routes to the inner ear include (1) RWM injection, (2) RWM injection combined with CF, (3) cochleostomy, (4) utricle injection, and (5) canalostomy. **(B)** Structure organ of corti. ASCC, anterior semicircular canal; PSCC, posterior semicircular canal; LSCC, lateral semicircular canal; RWM, round window membrane; CF, semicircular canal fenestration.

The inner ear, located within the petrous part of the temporal bone, consists of two primary sensory organs: (1) the peripheral vestibular system, which includes the semicircular canals, utricle, and saccule, relays information about linear and angular movements of the head to aid in maintaining equilibrium; (2) the cochlea, crucial for auditory transduction, converts sound waves into electrical impulses. Structurally, the cochlea contains three fluid-filled compartments: scala vestibuli, scala media (cochlear duct), and scala tympani, each isolated by Reissner’s membrane and the basilar membrane. The stria vascularis, situated along the scala media’s lateral wall, is notably vascularized and actively transports potassium ions into the endolymph, thereby playing an essential role in establishing and maintaining the endocochlear potential (EP) (Figure 1B).

The organ of Corti, the auditory sensory apparatus, comprises a single row of inner hair cells (IHCs) and three rows of outer hair cells (OHCs), both accompanied by supporting cells (SCs) (Figure 1B). The apical stereocilia of these hair cells (HCs), equipped with mechanotransduction channels, are particularly susceptible to damage and are pivotal for auditory signal transduction. Hair cell depolarization prompts the release of neurotransmitters from synaptic vesicles, which subsequently activate spiral ganglion neurons (SGNs). These neurons convey electrical signals through the eighth cranial nerve to the cochlear nucleus in the brainstem, facilitating sound perception and interpretation within our environment.

## Overview of AAV

### General information on AAV and recombinant AAV

AAV belongs to the genus Dependoparvovirus with the Parvoviridae family. Its lifecycle is reliant on other helper viruses such as Adenovirus (Balakrishnan and Jayandharan, 2014). AAV’s structure is characterized by a 4.7 kb single-stranded DNA genome. It consists of *rep* and *cap* genes, which are flanked by inverted terminal repeats (ITRs) within a nonenveloped capsid that is icosahedral and has a diameter of 26 nm (Wang et al., 2019). ITRs are the only cis-acting elements required for genome replication and packaging (Wu et al., 2006). The *rep* gene encodes four proteins, namely Rep78, Rep68, Rep52, and Rep40. These proteins, required for viral replication, are named by their molecular masses (Wang et al., 2019). The *cap* gene encodes three structural proteins: VP1, VP2, and VP3, in a ratio of 1:1:10 and assemble to form viral capsid and are involved in cell binding and internalization (Samulski and Muzyczka, 2014). On the virion surface, each VP subunit has nine variable regions, which determine the primary tropism and intracellular trafficking of AAV vectors, and neutralizing antibodies typically recognize these domains (Xie et al., 2002; Govindasamy et al., 2006). Engineering these variable regions can increase cell transduction efficiency and escape from antibody neutralization (Kotterman and Schaffer, 2014; Büning et al., 2015).

Currently, four expression systems are used for recombinant AAV (rAAV) production: adenovirus, herpesvirus, baculovirus complementation, and yeast expression systems (Aponte-Ubillus et al., 2018). One of the most prevalent methods for rAAV production involves the transfection of HEK293 cells with triple plasmids in the adenovirus complementation system. This method necessitates the delivery of three components into the host cell line: (1) the AAV vector genome composed of the transgene of interest; (2) the *rep* and *cap* genes; (3) the helper genes from the adenovirus (Aponte-Ubillus et al., 2018; Penaud-Budloo et al., 2018; Verdera et al., 2020). The essential components, including the transgene itself and regulatory elements like the promoter, polyA, and introns, are inserted between the ITRs of the AAV vector in place of both *rep* and *cap* genes (Lu, 2004; Kotterman and Schaffer, 2014). Accordingly, the maximum packaging capacity of rAAV is approximately 4.7 kb. These modifications render rAAV replication-deficient, enabling it only to infect cells and deliver DNA to their nuclei. In the current herpesvirus-based design, AAV-rep/cap, the transgene, and HSV-helper elements are delivered to a mammalian cell line like BHK, utilizing two recombinant herpes strains (Aponte-Ubillus et al., 2018; Penaud-Budloo et al., 2018). The baculovirus expression system in insect cells shows significant potential as a platform for rAAV production, exhibiting high per-cell and volumetric productivity. In this system, the production and assembly of the rAAV vector occur in insect cells and require a recombinant baculovirus vector to express the *rep* and *cap* genes (Urabe et al., 2002; Aponte-Ubillus et al., 2018). In yeast-based systems, the expression of AAV proteins is regulated by natural yeast promoters by transforming a collection of plasmids containing six AAV expression cassettes and a transgene (Barajas et al., 2017).

The rAAV vectors are extensively utilized in gene therapy due to their non-pathogenic characteristics, minimal potential for inflammation, the availability of a variety of viral serotypes, and the infrequent integration into the host chromosome, which leads to long-lasting gene expression (Aponte-Ubillus et al., 2018; Li and Samulski, 2020). Notwithstanding these advantageous characteristics, studies have indicated that AAV vectors may initiate immune responses within the inner ear (Ishibashi et al., 2023). The immune reactions and related adverse effects directly correlate with the dosages of AAV vectors in systemic therapeutic trials. Moreover, existing research has demonstrated that higher doses of AAV vectors can enhance transduction efficiency in hair cells (Ivanchenko et al., 2021). In light of these findings, future research should be directed towards developing vectors that can transduce cells with greater efficiency, augment expression of the transgene product, or deliver transgenes of heightened activity (Ertl, 2022). Adopting these strategies could reduce the required dosage of AAV vectors.

### The AAV transduction pathway

The AAV vector is internalized into the cell via endocytosis. The process initiates with the attachment of the AAV vector to receptors and co-receptors on the target cell’s surface. Once attached, the AAV vector is enclosed in endosomes through Clathrin-coated pits (Bartlett et al., 2000). After internalization, conformational changes in the viral capsid expose the N-terminal domain of the VP1 and VP2 proteins, facilitating the escape of the AAV vector from the endosomes to reach the nucleus (Bleker et al., 2005; Kronenberg et al., 2005). Upon arrival in the nucleus, the AAV vector undergoes capsid uncoating, a process during which the viral capsid is disassembled, revealing the viral genome. Consequently, the single-stranded DNA genome of the AAV is transformed into a double-stranded DNA genome, serving as the template for transgene to be transcribed and translated (Wu et al., 2006; Hastie and Samulski, 2015; Li and Samulski, 2020). In AAV-based gene therapy, the single-stranded rAAV genome that carries the transgene can express specific exogenous recombinant proteins within the host cells through the transduction pathway.

### AAV serotypes used for inner ear gene therapy

In the early days of inner ear gene therapy, most AAV vectors were either natural serotypes of AAV (1–12) or their improved variants, each exhibiting different tropism and transduction potentials towards remote organs or tissues. The cochlea is a complex organ with various cell types responsible for its structure and function, including HCs, SCs, and SGNs. HCs are comprised of OHCs and IHCs, which are responsible for amplifying sound and mechanoelectrical transduction, respectively (Géléoc and Holt, 2014). Several groups have analyzed the tropism profile of frequently used AAVs for inner ear cell transduction (Askew and Chien, 2020; Bankoti et al., 2021). IHCs can usually be transduced highly efficiently by AAVs, including AAV1, 2, 5, 7, 8, 9, and rh.8. Conversely, most AAVs demonstrate low transduction efficiency towards OHCs, with AAV1, 2, and 8 reported to transduce OHCs (Shu et al., 2016). In a study in 2015, Zinn et al. (2015) deduced approximations of evolutionary capsid intermediates that yield infectious particles through ancestral sequence reconstruction. They identified Anc80 as the likely ancestor of AAV serotypes 1, 2, 8, and 9. Among these, Anc80L65 stood out as a highly potent *in vivo* gene therapy vector, with the ability to target the liver, muscle, and retina. Landegger et al. (2017) demonstrated that Anc80L65 could effectively transduce OHCs and IHCs (95% OHCs and 100% IHCs transduction at the base of the cochlea) via round window membrane (RWM) injection, marking a substantial improvement over conventional AAV vectors. Deverman et al. (2016) designed a unique capsid selection method named Cre recombination-based AAV targeted evolution. They developed a library for AAV variants by inserting seven randomized amino acids (7-mer) between the VP1 site of the AAV9 capsid (amino acids 588 and 589). This resulted in the generation of an AAV variant, AAV-PHP.B, which is 40-fold more efficient than the current standard AAV9 at transferring genes throughout the central nervous system (Chan et al., 2017). Lee et al. (2020) reported a higher transduction efficiency in both OHCs and IHCs through utricle injection AAV9-PHP.B into the postnatal 7 days (P7) and P1 mice, compared to mice injected with Anc80L65. Initially, gene therapy vectors for hereditary HL mouse models were mainly based on AAV1, AAV2, and their variants (Akil and Lustig, 1950; Isgrig et al., 2017; Dulon et al., 2018). However, following the discovery of the high transduction rate of Anc80L65 and AAV9-PHP.B targeting HCs, these engineering AAV vectors have been increasingly used in gene therapy research of hereditary HL (Pan et al., 2017; György et al., 2019a; Taiber et al., 2021; Wu J. et al., 2021).

Gene therapy mediated by AAV shows promise not only in treating hereditary HL but also in addressing acquired HL, by facilitating the delivery of therapeutic gene cDNA to the cochlea for the regeneration of inner ear stem cells. Lgr5, Axin2, and Frizzled9-positive cells in the inner ear auditory epithelium have been identified as inner ear stem cells (Chai et al., 2012; Jan et al., 2013; Zhang et al., 2019). Lgr5, a downstream target gene of the Wnt signaling pathway, is expressed in specific SCs of the mouse cochlea, including in the third-row Deiters’ cells, inner pillar cells, inner phalangeal cells, and lateral greater epithelial ridge cells (Chai et al., 2011, 2012). Lineage labeling has shown that Axin2-positive cells are located at tympanic border cells, and Frizzled9 is expressed in inner phalangeal cells, inner border cells, and third-row Deiters’ cells of the mouse (Jan et al., 2013; Zhang et al., 2019). In damaged neonatal mouse utricles, Lgr5-positive cells have shown capabilities for regenerating HCs through proliferation and direct transdifferentiation (Wang et al., 2015).

Given that inner ear Lgr5-positive SCs are inner ear stem cells, evaluating the transduction rate of AAVs targeting SCs is critical for gene therapy aimed at hair cell regeneration, and several research groups have undertaken this work (Shu et al., 2016; Gu et al., 2019; Hu et al., 2019; Isgrig et al., 2019). Gu et al. (2019) demonstrated that the transduction efficiency of AAV2/9 in SCs post *in vivo* injection is 21.9%, and AAV2/Anc80L65 exhibits a higher efficiency of nearly 30%. Hu et al. (2019) reported that the transduction efficiency of AAV-DJ in SCs is approximately 50% through RWM injection of mice. AAV2.7m8 infects Lgr5-positive SCs (inner pillar cells and inner phalangeal cells) with high efficiency, respectively, 86.1 ± 4.56% and 61.4 ± 9.30% at the middle turn of the cochlea. However, it rarely infects Deiters’ cells, which are crucial for hair cell regeneration (Cox et al., 2014; Isgrig et al., 2019). Therefore, further optimization of AAV to improve its transduction efficiency for all types of inner ear stem cells has become a critical technical challenge. Based on AAV-DJ, a more efficient and safer variant, AAV-ie, was developed to address this problem. AAV-ie, has a transduction rate exceeding 90% for both OHCs and IHCs. At an equivalent dosage, Anc80L65 and AAV-DJ achieved moderate transduction efficiencies in SCs of less than 55%, whereas AAV-ie demonstrated a markedly higher efficiency, transducing SCs at approximately 77% (Tan et al., 2019). Notably, AAV-ie’s transduction of SCs exhibited a dose-dependent relationship. At higher doses, AAV-ie efficiently targeted all subtypes of SCs, including Hensen’s cells, Deiters cells, pillar cells, inner phalangeal cells, and inner border cells, with the overall efficiency surpassing 80% (Tan et al., 2019). Moreover, the delivery of the *Atoh1* gene into the mouse cochlea by AAV-ie generates numerous new HC-like cells (Tan et al., 2019). In 2022, the same research group performed mutational screening on AAV-ie capsid and reported that a particular amino acid-mutant AAV-ie capsid, AAV-ie-K558R, has higher transduction efficiency in both the OHCs located in the middle region of cochlea and the SCs located in the basal region (Tao et al., 2022). When AAV-ie-K558R was used to deliver *Prestin*, a motor protein of OHCs that plays a vital role in mediating their electromotility, to P1-P2 *Prestin* KO mice, it partially restored the auditory brainstem response (ABR) threshold with more pronounced effect in the high frequencies (Tao et al., 2022). Furthermore, AAV-ie-K558R has been shown to induce the regeneration of HC-like cells in the region of the greater epithelial ridge. Some cells develop kinocilium when *Atoh1* is delivered into the mouse cochlea via RWM at P3 (Tao et al., 2022). These studies suggest that AAV is a promising vector for restoring HL caused by genetic dysfunction and rescuing HL caused by noise-induced injury, ototoxic drugs, or aging through hair cell regeneration.

### Dual-AAV system and dual-AAV application in the treatment of hereditary HL

As mentioned earlier, the packaging capacity of AAV is approximately 4.7 kb. Several strategies have been explored to enhance AAV’s capacity to deliver larger genes. These include optimizing regulatory elements (such as promoters and polyA sequence), developing shortened versions of therapeutic genes that encode a truncated but functional protein, and manipulating AAV capsids. However, these efforts only marginally increase the capacity of AAV and seem insufficient for transporting larger genes. These larger genes, such as those encoding otoferlin (OTOF, 6 kb), myosin 7A (MYO7A, 6.5 kb), myosin 15A (MYO15A, 10.6 kb), otogelin (OTOG, 8.8 kb) and otogelin-like (OTOGL, 7 kb), cadherin-23 (CDH23, 10 kb), or protocadherin-15 (PCDH15, up to 5.9 kb) (Reisinger, 2020), which is responsible for potential substantial patient population. The most well-studied and validated method to overcome the limited AAV cargo capacity is the dual-AAV system, which uses two vectors to carry two parts of one gene. This system has been successfully applied in preclinical and clinical trials (Patel et al., 1950; Tornabene and Trapani, 2020). The dual-AAV system can reconstitute the large therapeutic proteins at the DNA, pre-mRNA, or protein level.

**Figure 2:**
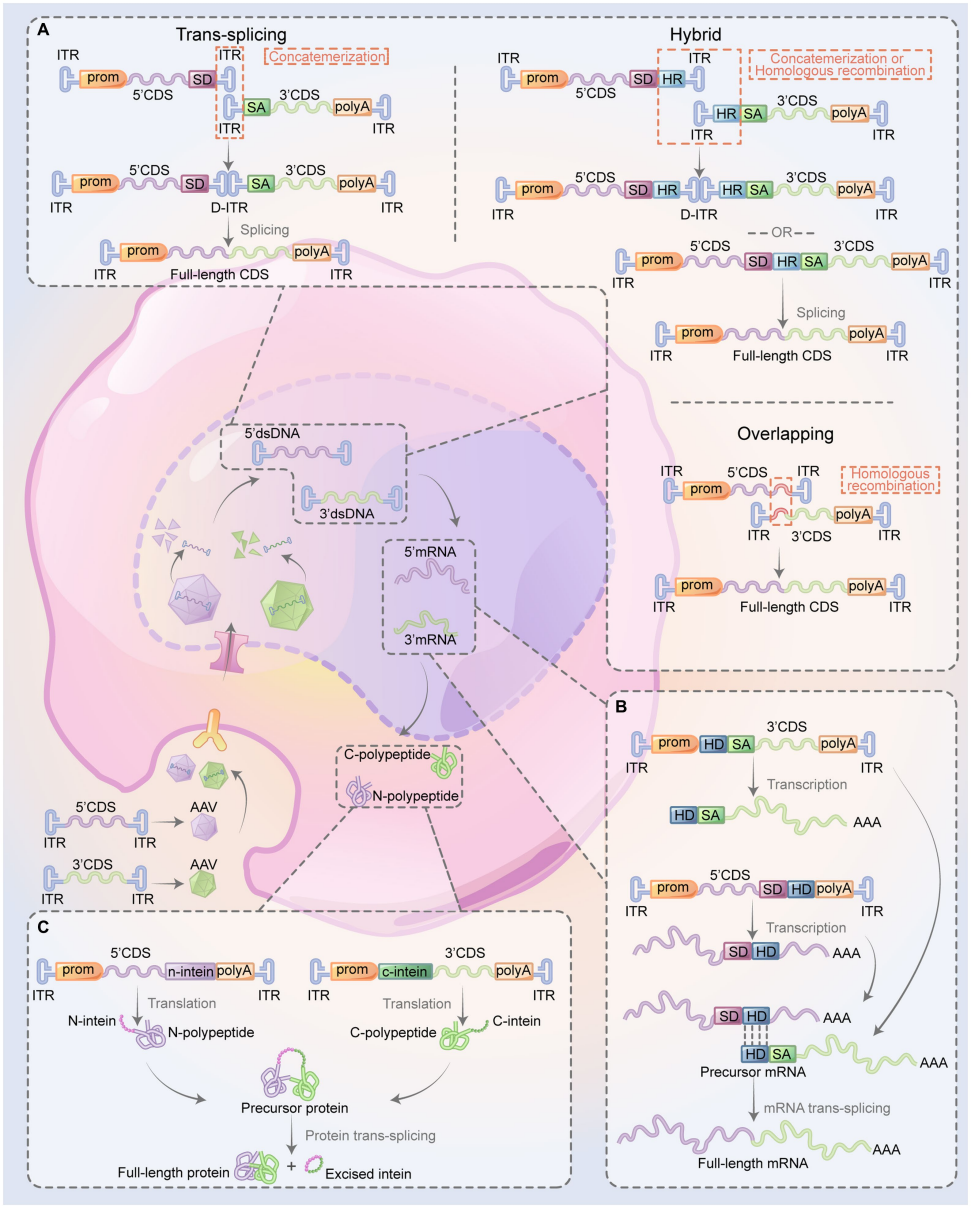
Overview of dual-AAV system restoring the expression of the large therapeutic proteins. The CDS of a gene is bifurcated into two sections (5’ and 3’), each of which is encapsulated into two AAVs. When co-transduced into the same cell, various methods are be used to reestablish the expression of large therapeutic proteins. This restoration process involves the recombination of the two segments at multiple stages: the DNA level **(A)**, the pre-mRNA level **(B)**, or directly at the protein level **(C)**. **(A)** Reconstitution full-length CDS: the trans-splicing method involves the reconstitution of the full-length CDS through the process of concatemerization of the two vectors mediated by ITRs, followed by the splicing signals which eliminate the ITR structure. In the overlapping method, both vectors contain an overlap region, which functions as a homologous sequence, enabling the two gene segments to recombine effectively. The hybrid method design of the two vectors imitates that of the trans-splicing method, but with an added HR positioned downstream of the SD in the 5’-vector and upstream of the SA in the 3’-vector. The full gene can be reassembled either through ITR-mediated concatemerization or homologous recombination, followed by splicing to remove the intermediate sequence. **(B)** Reconstitution of full-length mRNA: in this approach, the two mRNA pair up via their complementary HDs, followed by splicing which results in the elimination intermediate sequence. **(C)** Reconstitution of full-length protein: the self-recognition and self-excision of inteins facilitate pairing the two half polypeptides to form the full-length protein. 5’CDS, 5’ portion of the coding sequence; 3’CDS; 3’ portion of the coding sequence; AAV, adeno-associated virus; ITR, inverted terminal repeat; HR, highly recombinogenic exogenous sequence; SD, splicing donor; SA, splicing acceptor; HD, hybridization domain; prom, promoter; polyA, polyadenylation (figure modified from Tornabene and Trapani, 2020).

Reconstitution of DNA using dual-AAV strategies relies on different mechanisms: trans-splicing, overlapping, and hybrid AAV vectors (Figure 2A). The trans-splicing method exploits the inherent concatamerization ability of AAV’s ITRs to reassemble full-length genomes (Yan et al., 2000). The 5′-vector carries the promoter, the 5′-portion of the coding sequence (CDS), and a splicing donor (SD) signal, whereas the 3′-vector carries a splicing acceptor (SA) signal, the 3′-portion of the CDS, and the polyA signal. When ITR-mediated concatamerization links the two AAV vector genomes in a head-to-tail orientation, the complete gene expression cassette is reinstated if splicing signals are triggered to remove the intervening recombinant ITR sequence (Nakai et al., 2000; Sun et al., 2000; Yan et al., 2000; Reich et al., 2003). The overlapping method relies on the homologous recombination within the CDS in both vectors to reconstitute the intact gene expression cassette (Duan et al., 2001). The 5′-vector contains the promoter, the 5′-portion of the CDS, whereas the 3′-vector has the 3′-portion of the CDS and the polyA signal. The CDS in both vectors shares a homologous region. The hybrid AAV method leverages both trans-splicing and overlapping mechanisms (Patel et al., 1950; Ghosh et al., 2008; Tornabene and Trapani, 2020). It is anticipated to have higher recombination efficiency because it incorporates a highly recombinogenic exogenous sequence, such as a region from the alkaline phosphatase gene or the phage F1 genome, into the trans-splicing vectors. In the 5′-vector, an SD signal is engineered upstream of the recombinogenic sequence, while in the 3′-vector, an SA signal is incorporated downstream of the recombinogenic sequence. The gene cassette can be reconstituted via ITR-mediated concatamerization rejoin of the two AAVs or via recombinogenic sequence-mediated homologous recombination. Subsequently, the splicing signal removes the recombinogenic sequence and/or recombinant ITR structure to restore the gene expression (Ghosh et al., 2008). Compared to the overlapping AAV method, where efficiency relies on both the transgene and the target cell, the hybrid AAV method does not require a homologous recombination sequence in the CDS. This feature allows the hybrid AAV method to have broader applicability, but it also might induce an immunogenic response due to the introduction of foreign DNA elements. Regarding the head-to-tail orientation, which is the speed-limiting process for the trans-splicing method, the hybrid AAV method can enhance the correct direction of the two vectors to improve recombination efficiency (Ghosh and Duan, 2007).

Trans-splice-mediated pre-mRNA rejoining to form complete mRNA involves a mechanism during the post-transcriptional processing of pre-mRNA in eukaryotic cells (Figure 2B). This concept is based on the “segmental trans-splicing” system developed by Pergolizzi et al. (2003). In this system, one vector contains 5’CDS, SD, and a hybridization domain (HD), while the other one contains HD, a branch point, an SA, and the 3’CDS. This arrangement gives rise to pre-mRNA containing 5′- and 3′- splice signals. Both pre-mRNAs carry complementary HDs, which allows them to interact and facilitates the trans-splicing of the two mRNA fragments, thereby reconstituting a complete mRNA (Pergolizzi et al., 2003). Through this strategy, delivering the 5′- and 3′- halves of the cystic fibrosis transmembrane conductance regulator (CFTR) cDNA by two AAV vectors could enable the splicing of two pre-mRNAs, forming a complete and functional CFTR mRNA in human cystic fibrosis airway epithelial IB3-1 cells (Song et al., 2009).

At the protein level, the dual-AAV system restores full-length protein expression by utilizing split-inteins, which are protein segments with the remarkable ability to self-splice and rejoin to form a functional protein (Figure 2C). In the dual-AAV system, split-inteins contain two separate fragments, namely the N-intein and C-intein, engineered at the end of the target protein’s 5′ and 3′ halves, respectively. When the N-intein and C-intein fragments are brought into proximity, they rapidly self-assemble, excising the intein sequence and ligating the flanking protein sequences, resulting in the formation of a full-length functional protein (Wu et al., 1998).

In ongoing preclinical and clinical trials, the dual-AAV system can be utilized to deliver large genes, CRISPR/Cas9 system, or a base editor, based on diverse gene therapy strategies (Al-Moyed et al., 2019; Akil et al., 2019b; Yeh et al., 2020; Shubina-Oleinik et al., 2021; Tang et al., 2023). The *OTOF* gene, expressed in the ribbon synapse vesicles of the inner ear, plays a crucial role in afferent synaptogenesis (Roux et al., 2006). In a study by Al-Moyed et al. (2019), dual-AAV2/6 vectors, each carrying half of the *Otof* cDNA, were coinjected into the cochlea of P6 and P7 *Otof*^−/−^ mice through RWM, resulting in the successful expression of full-length OTOF in approximately 50% of IHCs and the observed complete restoration of fast exocytosis in IHCs with dual-AAV transduction. Akil et al. (2019b) used dual AAV2 quadY-F vectors containing 5′- and 3′- portions of *Otof* cDNA. These vectors were delivered into the cochlea of *Otof*^−/−^ mice at different stages: P10 (before hearing onset), P17 (before IHCs ribbon synapses maturation), and P30 (mature cochlea) through RWM. All treated mice restore ABR within 10 dB of wild-type (WT) mice. Consequently, hearing thresholds in response to clicks remained the same until 20–30 weeks post-injection. A reversal of the deafness phenotype and the long-lasting restoration of hearing thresholds in the mature stage of *Otof*^−/−^ mice provide promising prospects for treating autosomal recessive deafness (DFNB)-9 patients with *OTOF* deficiency (Akil et al., 2019b). More recently, Tang et al. (2023) restored complete OTOF expression using the split-inteins mechanism at the protein level, shifting from the DNA level reconstitution strategies used in the previous study. In this system, dual AAV-PHP.eB injected into the unilateral cochlea of *Otof*^−/−^ mice at P0-2 reversed bilateral deafness. Specifically, the hearing of treated mice was restored to nearly WT mice level at least 6 months, and IHCs reinstated the release of synaptic vesicles (Tang et al., 2023). Qi et al. (2023) pioneered a novel dual-AAV-OTOF-hybrid approach for delivering full-length OTOF, successfully restoring stable hearing in adult *Otof*^p.Q939*/Q939*^ mice suffering from profound deafness. Furthermore, they evaluated the transduction efficiency and safety of dual-AAV-OTOF in nonhuman primates (NHPs), providing crucial systematic research data to advance clinical investigations for DFNB9 patients (Qi et al., 2023). Based on these studies, clinical trials for dual-AAV-mediated gene therapy targeting *OTOF* have commenced. Four companies, Akouos, Decibel Therapeutics, Sensorion and RRGENER Therapeutics, are actively developing their respective *OTOF* gene therapeutic agents (Buck et al., 2022; Jiang et al., 2023; Shi et al., 2023). Shu et al. (2016) and Decibel Therapeutics/Regeneron have recently unveiled encouraging clinical findings, demonstrating safety and efficacy in trials involving DFNB9 patients. Significantly, Xue and colleagues surpassed the utilization of dual-AAV vectors for OTOF expression by introducing an enhanced mini-dCas13X RNA base editor using an AAV9 variant, resulting in the successful restoration of auditory function in *Otof*^Q829X/Q829X74^. Additionally, Shubina-Oleinik et al. (2021) developed the dual AAV9-PHP.B system to recover the exogenous STRC expression in OHCs of *Strc*^−/−^ mice, which led to the restoration of hair bundle morphology and cochlear amplification function. Apart from reconstituting protein expression encoded by large deficient genes based on the gene replacement strategy, a dual-AAV system can also accomplish genetic therapeutic effects from the perspective of gene editing. For instance, Yeh et al. (2020) packaged a cytosine base editor (CBE) into a dual AAV using split-intein to reconstitute a functional, full-length base editor. The inner ears of Baringo mice, which mimic DFNB7/11 in humans, were injected with the dual AAV at P1. Treated mice exhibited up to a 51% reversal of the *Tmc1c*.A545G point mutation to the WT sequence c.A545A, restoring inner hair cell sensory transduction, hair cell morphology, and partial auditory function (Yeh et al., 2020).

## Gene therapy strategies for SNHL

Implementing gene therapy strategies for different types of SNHL necessitates a personalized approach that thoroughly considers the distinct pathologies and molecular mechanisms specific to each underlying etiology.

Gene replacement is a fundamental strategy in gene therapy that addresses genetic disorders by introducing functional gene copies into cells to compensate for defective genes. While the potential of this approach is promising due to its straightforward design, several challenges remain. Achieving precise levels and timing of gene expression is crucial, as overexpression may lead to toxicity, while under-expression may diminish therapeutic efficacy. Furthermore, packaging large genes into a single AAV can be challenging.

Gene suppression selectively downregulates the expression of genes associated with disease mutations at the post-transcriptional level, often achieved through RNA interference (RNAi) and antisense oligonucleotides (ASOs) (Ghosh and Tabrizi, 2017). ASOs are short synthetic nucleic acid molecules that bind to complementary RNA sequences via Watson-Crick base pairing, leading to outcomes such as mRNA molecule degradation through the activity of cellular enzymes (RNase H), RNA splicing inhibition, or protein translation blocking (Robillard et al., 2022). Unlike gene replacement, using ASOs as a therapeutic intervention preserves the integrity of endogenous gene expression regulatory mechanisms. To date, the application of ASOs in hereditary HL mouse models has been based on a splice-switching mechanism. This approach forms a steric block at regulatory elements for cis-splicing, consequently changing how the splice site is recognized and promoting alternative splicing. This technique has successfully treated mice suffering from *Ush1c* c.216A point mutations that disrupt normal splicing (Lentz et al., 2013; Ponnath et al., 2018; Lentz et al., 2020). Another gene suppression approach, RNAi, uses small interfering RNAs (siRNAs), microRNAs (miRNAs), or CRISPR/Cas13 RNA editing system to bind to target mRNA, leading to its knockdown (Zheng et al., 2022). However, RNAi therapy faces many significant challenges, including potential off-target effects, immune responses leading to toxicity, the vulnerability of siRNA to rapid degradation, and the necessity of repetitive dosing (Ranasinghe et al., 2022).

Gene editing could modify or correct genetic mutations, employing tools such as the CRISPR/Cas9 system and base editors. Recently, RNA base editors, which correct RNA without cutting DNA, have been applied in inner ear gene therapy. In the CRISPR/Cas9 system, inducing double-strand breaks (DSBs) in target DNA activates the cell’s innate repair mechanisms. Two primary repair processes exist: non-homologous end joining (NHEJ) and homology-directed repair (HDR). NHEJ, although an efficient process, may lead to insertions or deletions (INDELs) at the DNA break site, potentially causing frameshift mutations and creating knock-out mutant genes. On the other hand, HDR, when a donor DNA template is available, allows for precise genetic modifications like correcting a disease-causing mutation or incorporating a therapeutic gene (Kang et al., 2019). The advantage of base editors lies in their ability to precisely modify individual nucleotides without inducing DSBs or relying on donor DNA templates, as required by CRISPR/Cas9 system. This precision makes them especially useful for correcting point mutations. The most commonly used base editor is the CBE, which facilitates the conversion of cytosine (C) bases to thymine (T). Another type is the adenine base editor (ABE), enabling the transformation of adenine (A) bases to guanine (G) by cellular machinery (Komor et al., 2016). However, it’s important to note that a more comprehensive evaluation of the impacts of gene editing is necessary, particularly concerning off-target effects, deletions, and rearrangements. The consequences of these events remain unclear (Davies, 2019).

Gene addition introduces a therapeutic gene, often playing a role in hindering the degeneration of inner ear neurons or promoting the regeneration of auditory HCs in SNHL gene therapy.

## Application of AAV-mediated gene therapy in the hereditary HL

Most identified causes for hereditary HL are attributed to defects in a single gene. Determining of a specific gene therapy system demands thorough consideration of factors such as the pattern of inheritance, the cellular mechanisms of the gene within the inner ear, the route of AAV delivery, and the intervention time window. The following section will discuss the successful applications of gene therapy in the context of hereditary HL.

### Inheritance pattern and pathogenic mechanism of hereditary HL

Hereditary HL falls into two categories: non-syndromic HL (NSHL) and syndromic HL (SHL). NSHL is characterized by isolated hearing impairment or total HL without additional abnormalities or syndromic features. On the other hand, SHL is HL that occurs as part of a syndrome. NSHL can be further classified according to its inheritance pattern: autosomal dominant (DFNA), DFNB, X chromosome-linked (DFNX), and mitochondrial. The most prevalent form of NSHL is the autosomal recessive pattern, accounting for 75%–80% of cases. About 20% of cases are due to autosomal dominant form. In contrast, X-linked and mitochondrial inheritance patterns are less commonly observed, accounting for less than 2 and 1% of cases, respectively (Sheffield and Smith, 2019). To date, there are 157 genetic loci with 110 identified genes (Sheffield and Smith, 2019). Almost 80% of all cases of prelingual hereditary HL are caused by DFNB forms, while post-lingual and late-onset HL is generally characteristic of DFNA forms. Autosomal dominant disorders primarily manifest through three mechanisms: haploinsufficiency, dominant negative effects, and gain-of-function (GOF) effects. In the case of haploinsufficiency, the mutant allele produces an abnormal or nonfunctional protein, and the single normal allele cannot compensate for this deficiency. Dominant negative effects occur when the mutant protein interferes with the function of the normal protein encoded by the unaffected allele. In the mechanism of GOF effects, the mutation allele leads to new or enhanced protein function. Unlike dominant disorders requiring only one mutated allele, recessive disorders occur when both alleles carry loss-of-function (LOF) variants. These variants can be missense, nonsense, splicing variations, copy number variants, and INDELs that compromise the protein’s function.

Given the variety of genetic mechanisms and inheritance patterns, the selection of gene therapy strategies is accordingly diverse. In general terms, gene replacement therapy could be advantageous for either dominant disorders that result from a haploinsufficiency mechanism or recessive disorders. Recessive disorders caused by splicing variations and dominant disorders with haploinsufficiency might benefit from ASOs, which correct abnormal pre-mRNA splicing to restore adequate levels of functional protein. RNAi can be used in dominant disorders characterized by dominant negative effects or GOF, disrupting the target mRNA encoded by the mutated allele. The CRISPR/Cas9 system, which disrupts DNA through the NHEJ mechanism, can also be employed to manage dominant negative effects or GOF mutations. Moreover, given the CRISPR/Cas9 system’s ability to induce INDELs through NHEJ, this strategy can be utilized to address frameshift mutations (Liu et al., 2022). The CRISPR/Cas9 system-mediated HDR, base editors, and RNA base editors are particularly suited for addressing point mutations, regardless of the inheritance pattern. Currently, gene addition therapy is primarily applied in cases of acquired HL, with limited research involving its application in hereditary HL mouse models.

### Target genes in gene therapy for hereditary HL

Understanding the cellular mechanisms of HL genes is crucial for the success of gene therapy. We summarize the HL genes successfully treated using AAV-mediated gene therapy in mouse models (Table 1).

**Table 1:** Display of cochlear genes successfully targeted by AAV-based inner ear gene therapy.

Gene	Target cell & function	Mouse model	Strategy	Age of intervention	AAV	Route	Outcome (auditory or morphology analysis)	Reference
*MYO6*	HCs/MYO6 anchors the stereocilia to the cuticular plate	*Myo6* ^*p*.*C442Y*/+^	Gene editing (CRISPR/Cas9 system-mediated NHEJ knockout)	P0–P2	AAV-PHP.eB	Scala media	Rescue of auditory function was observed for up to 5 months. More regular hair bundle morphology, and recovery of inward calcium levels	Xue et al. (2022)
			RNA base editors	P0–-P2	AAV-PHP.eB	Scala media	Rescue of auditory function up to 3 months. The increased survival rate of HCs and decreased degeneration of hair bundle morphology	Xiao et al. (2022)
*TMC1*	HCs/TMC1 affects the permeation properties of sensory transduction channels in HCs	*Tmc1* ^−/−^	Gene replacement	P0–P2	AAV2/1	RWM	ABR showed partial recovery of the hearing threshold; DPOAE shows no recovery	Askew et al. (2015)
		*Tmc1* Beethoven p.M412K	Gene replacement	P0–P2	AAV2/1	RWM	The delivery of AAV1-CBA-*Tmc2* improved inner HC survival	Askew et al. (2015)
		*Tmc1* ^−/−^	Gene replacement	P1–P2	AAV2/Anc80L65	RMW	Partial recovery of ABR thresholds, especially at the low frequencies	Nist-Lund et al. (2019)
		*Tmc1* ^−/−^	Gene replacement	P1, P7	AAV2/9-PHP.B	Utricle	Delivered at P1 can prevent HC loss and recover hearing, whereas P7 injections may be insufficient for full hearing recovery	Wu J. et al. (2021)
		*Tmc1* Baringo p.Y182C	Gene replacement	P1, P7	AAV2/9-PHP.B	Utricle	Delivered at P7 resulted in stronger recovery of both ABR and DPOAE thresholds compared to *Tmc1*^−/−^	Wu J. et al. (2021)
		*Tmc1* Beethoven p.M412K	Gene editing (CRISPR/Cas9 system-mediated NHEJ knockout)	P1	Dual AAV9-PHP.B	Utricle	Selectively disrupted a dominant Tmc1 allele and preserved hearing	Wu J. et al. (2021)
		*Tmc1* Beethoven p.M412K	RNAi (miRNA)	P0–P2	rAAV2/9	RWM	Significant preservation of hearing at 8 and 16 kHz. 32 kHz, no rescue	Shibata et al. (2016)
		*Tmc1* Beethoven p.M412K	RNAi (miRNA)	P15–P16, P56–P60, P84–P90	AAV2/9	RWM + CF	Partial recovery of hearing function at P15–P16 and P56–P60, whereas no restoration of hearing function at P84–P90	Yoshimura et al. (2019)
		*Tmc1* Beethoven p.M412K	RNAi (CRISPR/Cas13 RNA editing system)	P1–P2	AAV-PHP.eB	RWM	Ameliorated the auditory impairment with improved HCs survival rate and less hair bundle degeneration	Zheng et al. (2022)
		*Tmc1* Beethoven p.M412K	Gene editing (CRISPR/Cas9 system-mediated NHEJ knockout)	P1–P2	AAV2/Anc80L65	Inner ear	Prevented deafness up to 1 year post-injection	György et al. (2019b)
		*Tmc1* Baringo p.Y182C	Gene editing (cytosine base editor)	P1	Dual AAV2/Anc80L65	Inner ear	Restored inner HC sensory transduction and HC morphology and transiently rescued low-frequency hearing 4 weeks after injection	Yeh et al. (2020)
*PJVK*	HCs/PJVK is involved in the oxidative stress-induced proliferation of peroxisomes	*Pjvk* ^−/−^	Gene replacement	P3	AAV2/8	RWM	Partial restoration of ABR thresholds, normal ABR waveforms and wave amplitudes	Delmaghani et al. (2015)
		*Pjvk* ^*G292R*/*G292R*^	Gene replacement	P0–P1	Anc80L65	RWM	Substantial prevention of HI up to 3 weeks with decrease at 1 month; prevention of balance defects up to 4 months	Lu et al. (2022)
*MSRB3*	HCs/MSRB3 plays an important role in HC homeostasis	*Msrb3* ^−/−^	Gene replacement	E12.5	AA2/1	Otocysts	Preserved HCs, rescue morphology of stereociliary bundles, and normal ABR thresholds at both low and high frequencies at P28	Kim et al. (2016)
*USH1C*	HCs/USH1C is a scaffold protein that anchors the upper tip-link	*Ush1c* c.216G > A	Gene replacement	P0–P1, P10–P12	AAV2/Anc80L65	RWM	Rescue of auditory thresholds to within 20 dB of WT for low-to mid-frequency hearing at P0-P1, whereas no rescue of any auditory function at P10–P12	Pan et al. (2017)
*USH1G*	HCs/USH1G is important for proper HC function	*Ush1g* ^−/−^	Gene replacement	P2.5	AAV8	RWM	Rescue stereocilia bundle morphology. Partial hearing improvement appeared to be short-lived, with progressive hearing loss by approximately 12 weeks postnatally	Emptoz et al. (2017)
*WHRN*	HCs/WHRN is a scaffold protein that is important for stereocilia elongation	*Whrn* ^*wi*/*wi*^	Gene replacement	P4	AAV8	PSCC	Partial restoration of hearing for at least 4 months. Normal stereocilia bundles morphology	Isgrig et al. (2022)
		*Whrn* ^*wi*/*wi*^	Gene replacement	P1–P5	AAV8	RWM	Normal stereocilia length and bundle architecture were restored. Whirlin gene therapy also increased inner HC survival in the treated ears	Chien et al. (2016)
*LHFPL5*	HCs/LHFPL5 plays an important role in HC mechanotransduction by regulating tip-link assembly and channel conductance	*Lhfpl5* ^−/−^	Gene replacement	P1–P2	Exo-AAV1	RMW, cochleostomy	Both RWM and cochleostomy injection transduced with high efficiency both IHCs and OHCs. Cochleostomy also transduced SGNs and supporting cells. Hearing thresholds are improved at frequencies from 4 to 22 kHz	György et al. (2017)
*PCDH15*	HCs/PCDH15 strengthens the importance of protocadherin-15 in the morphogenesis and cohesion of stereocilia bundles	*Pcdh15^av-3J^*	Gene editing (CRISPR/Cas9 system-mediated NHEJ disruption)	P0–P2	AAV2/9	Scala media	Half of the mice gained improvements in auditory responses, and balance function was restored in the majority of injected mutant mice	Liu et al. (2022)
*TMPRSS3*	HCs/TMPRSS3 is a permissive factor for cochlear HC activation and survival upon the onset of hearing loss	*Tmprss3^A306T/A306T^*	Gene replacement	18.5 months	AAV2	RWM + CF	Rescue of the auditory function to a level similar to WT mice and rescue of the HCs and the SGNs	Du et al. (2023)
*NDP*	HCs/NDP is important for the development or maintenance of microvasculature and HCs	*Ndp^tm1Wbrg^*	Gene replacement	P2, P21, P30	AAV9	Systemic administration (intravenous injection)	P2 treatment prevented the death of the HCs	Pauzuolyte et al. (2023)
*CLRN1*	HCs/CLRN1 is critical for HC stereocilia bundle morphogenesis	*Clrn1* ^−/−^	Gene replacement	P1–P3	AAV2, AAV8	RWM	Delivery of AAV-smCBA-*Clrn1*-UTR did result in the delay of the progression of hearing loss, with the average hearing improvement of 38 dB SPL up to 150 days postnatally	Geng et al. (2017)
		*Clrn1*^*ex4*−/−^, cKO	Gene replacement	P1–P3	AAV2/8	RWM	10–20 dB threshold improvement in KO mouse, 30–40 dB improvement in cKO mouse. Hearing loss progressed over time	Dulon et al. (2018)
		*Clrn1* ^−/−^	Gene replacement	P1	AAV-S	RWM	Complete prevention of HI for low-range and mid-range frequencies up to 5 months	Ivanchenko et al. (2021)
		*Clrn1* ^−/−^	Gene replacement	P1	AAV-PHP.B	RWM	~20 dB threshold improvement at low frequencies. Best performers had 50 dB improvement	György et al. (2019a)
*VGLUT3*	IHCs/VGLUT3 encodes a glutamate transporter necessary for IHC synaptic transmission	*Vglut3* ^−/−^	Gene replacement	P1–P3, P10–P12	AAV2/1	RMW, cochleostomy	RWM at P1–P3, 100% of mouse recovered normal ABR thresholds	Akil et al. (2012)
		*Vglut3* ^−/−^	Gene replacement	4 weeks	AAV9-PHP.B	Cerebrospinal fluid administration	Restoring hearing, and the synapses between the HCs and the neurons are restored at the base of the cochlea base	Mathiesen et al. (2023)
		*Vglut3* ^−/−^	Gene replacement	5 weeks, 8 weeks, and 20 weeks	AAV8	PSCC	Auditory function was restored, and the hearing threshold remained stable for at least 12 weeks after rescue. The number of synaptic ribbons, as well as their morphology, was significantly recovered after gene therapy	Zhao et al. (2022)
*OTOF*	IHCs/otoferlin is expressed primarily in the inner HCs, and it is involved with synaptic vesicle reformation and exocytosis	*Otof* ^−/−^	Gene replacement	P6–P7	Dual AAV2/6	RWM	Partially rescued auditory function. Dual-AAV transduction of *Otof*^−/−^ IHCs fully restored fast exocytosis, while otoferlin-dependent vesicle replenishment reached 35%–50% of WT levels	Al-Moyed et al. (2019)
		*Otof* ^*Q829X*/*Q829X*^	Gene replacement	P0–P1	AAV9	RWM	Auditory function reaching similar levels as in WT mice	Xue et al. (2023)
		*Otof* ^−/−^	Gene replacement	P10, P17, P30	Dual AAV2 quadY-F	RWM	All treated mice restored ABR within 10 dB of the WT mouse, and hearing thresholds in response to clicks remained unchanged until 20–30 weeks post-injection	Akil et al. (2019b)
		*Otof* ^*p*.Q939*/Q939*^	Gene replacement	P2	Dual Anc80L65	RWM	Restoring stable hearing, with the longest duration being at least 150 days	Qi et al. (2023)
		*Otof* ^−/−^	Gene replacement	P5–P7	AAV-PHP.B	RWM	Partial prevention up to 5.5 weeks	Rankovic et al. (2020)
		*Otof* ^−/−^	Gene replacement	P0–P2	Dual AAV-PHP.eB	RWM	The hearing of the treated mice was restored to nearly WT mouse level at least 6 months, and IHCs reinstated the release of synaptic vesicles	Tang et al. (2023)
*KLHL18*	IHCs/KLHL18 is expressed in the stereocilia of IHCs	*Klhl18* ^*p*.*V55F/p*.*V55F*^	Gene editing (CRISPR/Cas9 system-mediated HDR)	P1	AAV9, AAV-PHP.eB	Inner ear	Ameliorated the auditory function of the mouse model with homozygous recessive mutations	Gu et al. (2022)
*CABP2*	IHC/CABP2 encodes calcium binding protein 2, a potent modulator of voltage-gated CaV1.3 channels in IHCs	*Cabp2* ^−/−^	Gene replacement	P5–P7	AAV2/1	RWM	Partial prevention of HI for up to 2 months	Oestreicher et al. (2021)
*STRC*	OHCs/STRC is important for hair bundles’ cohesion	*Strc* ^−/−^	Gene replacement	P0–P1	Dual AAV9-PHP.B	Utricle	Restoration of hair bundle morphology and cochlear amplification function	Shubina-Oleinik et al. (2021)
*SYNE4*	OHCs/SYNE4 is critical for nuclear positioning	*Syne4* ^−/−^	Gene replacement	P0–P1.5	AAV9-PHP.B	PSCC	Rescue of HC morphology and survival, nearly complete recovery of auditory function, and restoration of auditory-associated behaviors	Taiber et al. (2021)
*SLC26A5*	OHCs/SLC26A5 mediates OHCs electromotility	*Prestin knockout*	Gene replacement	P3	AAV-ie-K558R	RWM	Modest prevention of HI up to 4 weeks	Tao et al. (2022)
*KCNQ4*	OHCs/KCNQ4 is significant to K^+^ homeostasis	*Kcnq4* ^*c*.*827G* > *C*/+^	Gene editing (CRISPR/Cas9 system-mediated NHEJ knockout)	P0–P1	AAV2/Anc80L	Utricle, PSCC, RWM, scala media	It significantly improved auditory thresholds in auditory brainstem response and distortion-product of otoacoustic emission. It elevated KCNQ4 channel activity	Noh et al. (2022)
		*Kcnq4* ^*c*.*683G* > *A*/+^	Gene editing (CRISPR/Cas9 system-mediated NHEJ knockout)	P1–P2	AAV-PHP.eB	Scala media	Mice showed significantly lower ABR and DPOAE thresholds, shorter ABR wave I latencies, higher ABR wave I amplitudes, increased number of surviving OHCs, and more hyperpolarized resting membrane potentials of OHCs	Cui et al. (2022)
*GJB2*	SCs/GJB2 is responsible for gap junctions between adjacent cells	*Gjb2* cKO	Gene replacement	P0, P42	AAV1	RWM	Delivering the Gjb2 cDNA by AAV1 vectors through the RWM in P0, Gjb2 cKO mice successfully improved their hearing function and cochlear morphology, although mice treated at P42 had no improvement	Iizuka et al. (2015)
		*Gjb2* cKO	Gene replacement	P0–P1	AAV2/1	Scala media	Partial morphology recovery. ABR no recovery	Yu et al. (2014)
*KCNQ1*	SV/KCNQ1 plays a pivotal role in the transportation of K^+^ into endolymph and maintaining the EP	*Kcnq1* ^−/−^	Gene replacement	P0–P2	AAV1	Cochleostomy	Morphology showed that the collapse of Reissner’s membrane and degeneration of HCs and cells in the spiral ganglia were corrected. Auditory brainstem responses showed significant hearing preservation in the injected ears, ranging from 20 dB improvement to complete correction of the deafness phenotype	Chang et al. (2015)
*KCNE1*	SV/KCNE1 plays a pivotal role in the transportation of K^+^ into endolymph and maintaining the EP	*Kcne1* ^−/−^	Gene replacement	P0–P2	AAV1	PSCC	The early treatment prevented Reissner’s membrane and vestibular wall collapse, retained the normal size of the semicircular canals, and prevented the degeneration of inner ear cells. Could improve auditory function in a dose-dependent manner	Wu X. et al. (2021)
*SLC26A4*	Outer sulcus/SLC26A4 is a Cl/HCO3 anion exchanger that is critical for fluid homeostasis in the inner ear	*Slc26a4*^−/−^, *Slc26a4^tm1Dontuh/tm1Dontuh^*	Gene replacement	E12.5	rAAV2/1	Otocysts	Local gene delivery resulted in spatially and temporally limited pendrin expression, prevented enlargement, and failed to restore vestibular function but succeeded in the restoration of hearing. Restored hearing phenotypes included normal hearing as well as sudden, fluctuating, and progressive hearing loss	Kim et al. (2019)

AAV, adeno-associated virus; HC, hair cell; P, postnatal; RWM, round window membrane; DPOAE, distortion product otoacoustic emission; E, embryonal; ABR, auditory brainstem response; CF, semicircular canal fenestration; PSCC, posterior semicircular canal; IHC/OHC, inner/outer hair cell; cKO, conditional knockout; WT, wild type; SGN, spiral ganglion neuron; SC, supporting cells; SV, stria vascularis.

### Genes primarily impacting the hair cells

Given that over 50% of gene mutations responsible for hereditary HL affect HCs, gene therapy studies have primarily focused on these sensory cells (Ahmed et al., 2017). Numerous mouse models bearing mutations in genes associated with HCs’ stereocilia, including *Myo6*, *Tmc1*, *Ush1c*, *Ush1g*, *Whrn*, *Lhfpl5*, *Pcdh15*, *Clrn1*, *Klhl18*, and *Strc*, have shown promising results with the application of AAV-mediated gene therapies (Askew et al., 2015; Chien et al., 2016; Shibata et al., 2016; Emptoz et al., 2017; Geng et al., 2017; György et al., 2017; Pan et al., 2017; Dulon et al., 2018; Nist-Lund et al., 2019; Yoshimura et al., 2019; György et al., 2019a,b; Yeh et al., 2020; Shubina-Oleinik et al., 2021; Wu J. et al., 2021; Gu et al., 2022; Isgrig et al., 2022; Liu et al., 2022; Xiao et al., 2022; Xue et al., 2022; Zheng et al., 2022). *OTOF* and *VGLUT3* are mainly localized in IHCs and play a significant role in synaptic transmission. Gene therapy has been shown to partially restore auditory function in mouse models harboring mutations in these genes (Akil et al., 2012; Al-Moyed et al., 2019; Akil et al., 2019b; Zhao et al., 2022; Tang et al., 2023). In HCs, some specific genes, such as *SYNE4*, *CLDN14*, *TJP2*, *MYH14*, function in hair cell adhesion and maintenance (Delmaghani and El-Amraoui, 2020). Taiber et al. (2021) achieved rescue of hair cell morphology and nearly complete recovery of auditory function in *Syne4* gene knockout mice cochlea by delivering normal copies of the *Syne4* gene into the inner ear using AAV9-PHP.B.

### Genes primarily impacting the stria vascularis

Defects in ion channel proteins within the stria vascularis can disrupt the EP, resulting in HL. For instance, mutations in genes like *KCNQ1* and *KCNE1* can cause a hereditary disorder named Jervell and Lange-Nielsen syndrome (JLNS). Both congenital deafness and an increased risk of life-threatening heart arrhythmias characterize this syndrome. Studies have shown that cochleostomy injection of AAV1 containing *Kcnq1* into *Kcnq1* knockout mice resulted in significant hearing restoration, ranging from a 20 dB improvement to a complete correction of the deafness phenotype (Chang et al., 2015). Furthermore, the application of exogenous expression of KCNE1 in JLNS mouse models has been studied, revealing its potential to prevent the degeneration of inner ear cells and enhance auditory function in a dose-dependent manner (Wu X. et al., 2021).

### Genes primarily impacting the SCs

Surrounding and interacting with the HCs are SCs, which serve a diverse set of functions, including maintaining the structural integrity of HCs, regulating ions and small molecules homeostasis, and modulating the extracellular matrices, all of which are integral for the optimal functioning of the hair cells (Wan et al., 2013). Gap junctions constitute intercellular channels formed by connexin proteins that enable the direct communication and exchange of ions and small molecules between supporting cells. Consequently, it is not unexpected that mutations in connexin genes, like *GJB2* and *GJB6*, can lead to HL. The *SLC26A4* gene is mainly expressed in spiral prominence and external sulcus cells, affecting cochlea’s ion homeostasis (Everett et al., 1999). According to some estimates, over 60% of genetic HL cases are attributed to mutations in the *GJB2* and *SLC26A4* genes (Zhang et al., 2018). Gene therapy targeting these genes could potentially benefit a significant number of patients. However, findings from mouse models indicate that the intervention window is limited. For instance, administering AAV1 vectors containing the *Gjb2* cDNA via the RWM in P0 *Gjb2* cKO mice significantly enhanced their hearing function and the morphology of the cochlea. In contrast, mice treated at P42 showed no improvement, suggesting that early intervention is crucial for achieving positive outcomes in gene therapy for hearing-related conditions associated with these genes (Iizuka et al., 2015). Gene therapy aimed at targeting the *Slc26a4* null mutation in fetal mice has been observed to partially reversed hearing phenotypes and prevent enlargement of the vestibular system (Kim et al., 2019).

## Delivery route for mouse models

Mouse models mimicking human HL are commonly used to evaluate gene and pharmacological therapies. The delivery of AAV to the inner ear of mouse models for gene therapies typically encompasses local, systemic, and fetal administration methods. Furthermore, researchers have investigated the potential of AAV delivery via the cerebrospinal fluid to target the inner ear.

### Local administration

The established local delivery routes to the inner ear include: (1) RWM injection; (2) RWM injection combined with semicircular canal fenestration (CF); (3) cochleostomy; (4) utricle injection; (5) canalostomy (Figure 1A). The RWM approach is frequently used in clinical settings for cochlear implantation and remains the dominant approach for introducing gene therapy agents into the inner ear in mouse models. Furthermore, efficient transduction of HCs in NHPs was achieved through the RWM injection delivery of AAV9-PHP.B (György et al., 2019a). However, a drawback of the RWM approach is the uneven distribution of the viral vector throughout the cochlear duct, leading to base-to-apex gradient transduction in adult mice (Yoshimura et al., 2018). To overcome this, Yoshimura et al. (2018) combined RWM with CF, where the fenestration acts as a vent, facilitating longitudinal flow throughout the cochlea and ensuring a more uniform distribution of the injected vector. All treated mice displayed transient vestibular dysfunction, which resolved the following day (Yoshimura et al., 2018). In NHPs, this procedure can be replicated through RWM injection by creating an oval window fenestra, resulting in significantly increased transduction rates for inner ear HCs (Andres-Mateos et al., 2022). In future translation studies, it is crucial to assess whether this double opening of the cochlea affects the vestibular and auditory function of NHPs, as this information is vital for the success of clinical therapies. Cochleostomy is performed by creating a hole in the bony labyrinth, typically between the round window and basal turn of the cochlea, to enable the delivery of therapeutic vectors directly into the scala media. This approach is more invasive than RWM injection, resulting in comparable viral infection efficiencies for hair cells but causing permanent HL in adult mice, particularly at high frequencies (Chien et al., 2015). This method may be advantageous for achieving a higher transduction rate when the therapy target cell is located in the endolymph, such as stria vascularis cells. Lee et al. (2020) reported the utricle injection method in neonatal mice, resulting in a nearly 100% transduction rate in hair cells without causing any damage to auditory or vestibular functions. This approach is more suitable for delivering vectors into the inner ear of mice with hearing and balance defects. However, the facial nerve covering the utricle region makes this method challenging in clinical practice. The canalostomy approach involves performing a fenestration in the semicircular canal, usually the posterior canal, through which the vector is injected. Although this approach successfully delivers the vector in preclinical trials, inducing efficient transduction of hair cells without causing auditory damage, one of the disadvantages is that accurate injection is challenging, resulting in the vector being delivered into the endolymph and/or perilymph, depending on the orientation of the cannula (Suzuki et al., 2017).

### Systemic administration

The AAV vector is introduced into the bloodstream through intravenous injection in systemic administration. The study on WT neonatal mice using intravenous injection of rAAV2/9 has shown successful transduction on both sides of IHCs (with a transduction rate of 96% in the apical turn), SGNs, and vestibular hair cells. Importantly, hearing acuity remains unaffected in treated mice when assessed at P30 (Shibata et al., 2017). Notably, the blood-labyrinth barrier in rodents, fully established by P14, acts as a protective barrier, restricting the entry of therapeutic molecules from the vasculature into the inner ear fluids (Nyberg et al., 2019). This characteristic offers a broader therapeutic window for the systemic administration of treatments in mouse models. However, it is important to note that systemic administration requires a high dose, which could potentially increase the risk of off-target effects and toxicity.

### Fetal administration

Fetal administration primarily involves delivering bioactive agents into the fetal mouse otic vesicle and amniotic cavity. Otic vesicle delivery offers the potential for sustained bioactive agents near otic progenitor cells. In contrast, amniotic cavity delivery allows the treatment of the entire embryo with a bioactive agent (Hastings and Brigande, 2020). Multiple studies have suggested that AAV-mediated gene transfer to the fetal mouse otic vesicle can restore hearing in congenitally deaf mouse models lacking SLC26A4 or MSRB3 (Dulon et al., 2018; Kim et al., 2019).

### Cerebrospinal fluid administration

Blanc et al. (2021) administered various AAV serotypes (AAV2/8, AAV2/9, AAV2/Anc80L65) through cisterna magna injection, resulting in high-efficiency binaural transduction affecting nearly all IHCs in a basal-to-apical pattern, as well as a substantial population of SGNs in the cochlea’s basal region. Remarkably, this intervention had no adverse effects on auditory function or cochlear structures (Blanc et al., 2021). Further, Mathiesen et al. (2023) investigated the potential of delivering gene therapy via the cerebrospinal fluid to restore hearing in adult deaf mice. Through intracisternal injection of AAV carrying *Slc17A8*, they successfully rescued hearing in adult deaf Slc17A8^−/−^ mice (Mathiesen et al., 2023).

## Application of AAV-mediated gene therapy in acquired HL

Gene therapy for acquired HL utilizes a gene addition strategy involving the delivery of neurotrophic factors or other protective factors (like anti-apoptosis agents) to prevent the loss of cochlear sensory cells. Additionally, gene therapy may involve providing transcription factors (like Atoh1) to manipulate the cell cycle pathway and induce hair cell regeneration. This approach is not necessarily gene-specific and instead relies on our understanding of the interplay of crucial biological pathways essential for normal cochlear functions.

### Hair cell regeneration

Unlike certain non-mammalian species, such as birds, mammals lack the spontaneous capacity for hair cell regeneration after damage (Stone and Cotanche, 2007). In recent years, researchers have strived to understand the underlying mechanisms of hair cell regeneration and explore potential therapeutic strategies for promoting regeneration in mammals. Central to these efforts is the focus on manipulating two pathways leading to hair cell regeneration—the Wnt/β-catenin and Notch pathways. Specifically, by inhibiting the Wnt/β-catenin pathway, there is a potential to induce the mitotic generation of new HCs. Despite these efforts, utilizing this pathway for hair cell regeneration has yielded limited success thus far. On the other hand, suppressing Notch or overexpressing atonal-1 (Atoh1) can trigger existing SCs to transdifferentiate into new HCs. However, it is essential to note that this approach relies on the presence of SCs for successful regeneration. Past studies have indicated that mouse atonal-1 (Math1) overexpression results in varying degrees of hair cell regeneration and hearing improvement in multiple animal models of HL (Lustig and Akil, 2019; Tan et al., 2019; Tao et al., 2022). Building upon these encouraging results, the CGF166 clinical trial employed an adenovirus to deliver the human atonal-1 (Hath1) gene into the cochlea of patients with HL. The trial aimed to assess the safety, tolerability, and potential efficacy of CGF166 as a treatment for HL. Partial data from the phase I/II clinical trial was available online on October 8, 2021. One critical consideration is that hair cell regeneration may not be suitable for hereditary HL, as the newly regenerated HCs may still carry the defective gene.

### Preservation of hair cells and SGNs

By introducing exogenous expression of biological factors that preserve neurons, we might theoretically hold the potential to improve the prognosis of acquired HL.

Noise-induced HL (NIHL) is a type of acquired HL caused by exposure to loud sounds, either from a solitary intense sound exposure (blast) or repeated exposure to loud noises over a prolonged period. Early noise exposure can induce reversible HL, where hearing thresholds may revert to normal levels over time. Yet, the synaptic connections between HCs and the auditory nerve fibers can suffer permanent damage, leading to a condition colloquially known as synaptopathy or hidden HL. This significant loss of synaptic contacts can precipitate ongoing and progressive degeneration of the nerve fibers, culminating in the death of the SGNs (Ma et al., 2019). Evidence indicates that neurotrophin-3 (NT-3) regulates ribbon synapse density in the cochlea (Wan et al., 2014). Remarkably, by introducing AAV-NT3 into the cochlea, Chen and colleagues managed to partially reverse the synaptic loss of guinea pigs after acoustic overexposure (Chen et al., 2018). However, Hashimoto et al. (2019) demonstrated that while AAV-mediated overexpression of NT-3 can mitigate noise-induced synaptic damage, forced overexpression may also inflict detrimental effects on HCs during cochlear overstimulation. In a recent development, Mukherjee et al. (2022) proposed an innovative approach using local magnetic targeting of AAV2 (quad Y-F)-BDNF, which successfully reversed the reduction in ABR wave I amplitude and cochlear synaptopathy following NIHL.

Ototoxic drugs potentially threaten the delicate structures of the inner ear, such as the HCs and auditory nerve fibers, which are integral for hearing. Some common examples of ototoxic drugs include specific antibiotics (like aminoglycosides), chemotherapy agents (like cisplatin and carboplatin), loop diuretics (like furosemide and bumetanide), and non-steroidal anti-inflammatory drugs (NSAIDs). AAV-mediated gene overexpression of NT-3, glial cell line-derived neurotrophic factor (GDNF), and activity-dependent neurotrophic factor 9 (ADNF-9) in animal cochlea has shown a protective effect against aminoglycoside-induced HL, mitigating cell damage (Yao et al., 2007; Liu et al., 2008; Zheng et al., 2013). Nevertheless, there are reports of overexpression of GDNF in newborn mice leading to HL, likely due to cochlear nucleus pathology (Akil et al., 2019a). High-temperature requirement protein A2 (HTRA2) functions as a pro-apoptotic factor by binding to the X-linked inhibitor of apoptosis protein (XIAP), thereby inducing apoptosis. Interestingly, Gu et al. (2021) employed AAV delivery of the CRISPR/Cas9 system to target and disrupt Htra2 expression, which resulted in the amelioration of apoptosis, promotion of hair cell survival, and restoration of hearing function in neomycin-induced HL mice. This groundbreaking study signifies the first successful use of CRISPR/Cas9 gene editing to prevent and treat acquired HL. Moreover, the delivery of XIAP via AAV to the cochlea has been demonstrated to protect against audiometric changes and hair-cell loss caused by cisplatin ototoxicity (Cooper et al., 2006; Jie et al., 2015). Looking ahead, future research should concentrate on carefully optimizing the timing and dosage of neurotrophic factor administration to achieve the desired therapeutic effect. It is paramount to balance providing sufficient support for cell survival and preventing excessive growth that could lead to tumor formation.

## Prospective considerations for the clinical translation process

AAV-mediated gene therapy is a rapidly advancing field with substantial potential for treating HL. This approach has shown considerable promise in preclinical animal models. Yet, translating these findings into human clinical applications presents numerous complexities that require careful consideration.

### Importance of NHPs trials for validating gene therapy

The safety and effectiveness of gene therapy in mouse models must be validated in larger animal trials, such as those involving NHPs. Before considering their application in humans, exploring the cell tropism of AAV within the cochlea of NHPs at various developmental stages is crucial (Petit et al., 2023). It is also recognized that the AAV displays distinct variations in gene expression across multiple species. An essential facet of preclinical research is the rigorous validation of the vector’s design, ensuring it precisely targets pathological cells, thereby promoting effective expression of the introduced transgene. Furthermore, NHPs yield pivotal data regarding the appropriate dosages for gene therapy. This information is essential for evaluating gene therapies’ immunogenicity and overall safety. Given the complexity of the inner ear’s structure, gene delivery methods proven effective in small rodents may not be directly applicable to human anatomy. Trials with NHPs are thus instrumental in refining the delivery routes for human application. Notably, there are no standardized surgical procedures for inner ear drug delivery in clinical practices before clinical trials focusing on the *OTOF* gene.

### Distinguishing interventions: severe congenital vs. delayed onset progressive HL

Severe Congenital HL is attributed to specific genetic mutations impacting the development or functionality of auditory structures within the inner ear. For such cases, early intervention, ideally before extensive deterioration in the organ of Corti, is considered crucial. However, a majority of proof-of-concept studies have demonstrated beneficial auditory outcomes primarily through neonatal gene replacement procedures conducted no later than P7 in mouse models that mimic human HL (Petit et al., 2023). Hearing is initially detected in mouse around P12.In contrast, human hearing is detected at 19 weeks gestational age (WGA), and the therapeutic window may close as early as 18 WGA (Hastings and Brigande, 2020). Additionally, the otic vesicle, a feasible intrauterine target in embryonic mice, forms at approximately 6 WGA in humans and has a length of just a few hundred microns, making it challenging to intervene (Hastings and Brigande, 2020).

This leads to formidable technical obstacles in fetal intervention and raises profound ethical considerations regarding the administration of high-risk gene agents to human fetuses. With HL being a non-life-threatening condition, the ethical implications of undertaking fetal interventions that pose substantial risks to both the pregnant individual and the fetus must be carefully evaluated. The central ethical question is whether the anticipated benefits of such interventions are sufficient to outweigh the potential risks involved. Cochlear implantation is likely to remain the predominant treatment modality for this type of HL. Looking ahead, a synergistic approach that integrates cochlear implants with gene therapy presents a promising avenue for enhancing therapeutic efficacy. This strategy could potentially leverage both technologies’ strengths: the immediate auditory benefit provided by cochlear implants and the long-term biological improvements offered by gene therapy. Such an integrated treatment paradigm may offer a comprehensive solution for addressing complex auditory impairments.

Gene therapy exhibits considerable promise for treating delayed onset progressive HL. Such conditions, often attributable to genetic factors emerging later in life, may benefit from an extended therapeutic window, allowing timely intervention before a significant auditory decline occurs. This extended window could also mitigate the risk of gene therapy agents dispersing into the brain parenchyma, a concern alleviated by the progressive occlusion of communication between the cochlear perilymph and cerebrospinal fluid in humans.

Preclinical research utilizing animal models has yielded encouraging outcomes in the application of gene therapy for delayed onset progressive HL. Furthermore, in cases of HL caused by ototoxic drugs or noise exposure, gene therapy has the potential to either induce regeneration of damaged auditory cells or provide protection against further degeneration. This multifaceted potential of gene therapy, combined with understanding the mechanisms of the delayed onset HL, positions it as a significant area of interest for future therapeutic development and clinical trials.

### Enhancing the production of AAV vectors

Despite the successful clinical implementation of AAV-based gene therapies, such as Luxturna for inherited retinal disease and Zolgensma for spinal muscular atrophy, the production of AAV vectors for clinical use still requires optimization, including scaling up production, ensuring high purity and rigorous quality control, maintaining consistent potency for effective cell transduction and gene expression, ensuring stability for storage and transport, and reducing costs. AAV-mediated inner ear gene therapy is a promising field with the potential to transform the treatment landscape of inner ear disorders. While significant challenges remain, ongoing research continues to push the boundaries of what is possible, bringing us closer to a future where HL can be effectively treated or even cured at the gene level.

## Author contributions

LL: Writing – original draft, Writing – review & editing. TS: Writing – original draft, Writing – review & editing. SL: Writing – review & editing. JQ: Writing – review & editing. YZ: Writing – review & editing.
